# Religiosity and COVID-19: Impact on Use of Remote Worship and Changes in Self-Reported Social Support

**DOI:** 10.3390/ijerph19169891

**Published:** 2022-08-11

**Authors:** Maghboeba Mosavel, Ariel Hoadley, Aderonke A. Akinkugbe, Dina T. Garcia, Sarah Bauerle Bass

**Affiliations:** 1 Department of Health Behavior and Policy, Virginia Commonwealth University, 830 East Main Street, P.O. Box 980149, Richmond, VA 23298, USA; 2 Department of Social and Behavioral Sciences, College of Public Health, Temple University, 1301 Cecil B. Moore Ave., Ninth Floor, Philadelphia, PA 19122, USA; 3 Division of Environmental Epidemiology, Department of Environmental Medicine and Public Health, Icahn School of Medicine at Mount Sinai, New York, NY 10029, USA

**Keywords:** COVID-19, social support, religious institutions, religious worship services, remote worship

## Abstract

**Objective**: This study examines associations between changes in the use of remote worship services and changes in the types of social support among religious adults during the COVID-19 pandemic. **Materials and Methods**: Cross-sectional, web survey data (*n* = 461; 15 May to 6 July 2020) were collected during the COVID-19 pandemic. Multinomial logistic regression models calculated unadjusted odds of increases and decreases of three types of perceived social support from before to during COVID-19 based on remote worship use. **Results**: Adults who initiated use of remote worship had lower odds of gaining social support for personal problems (OR: 0.38; 95% CI: 0.19, 0.79) and greater odds of reporting less ease of getting practical help from neighbors (OR: 1.77; 95% CI: 1.04, 3.02) compared to adults who never used or stopped using remote worship. Adults who continued using remote worship services were more likely to report less ease of getting practical help from their neighbors (OR: 2.23; 95% CI: 1.17, 4.25) and decreased interest and concern felt from other people (OR: 2.62; 95% CI: 1.24, 5.51) than adults who never used or stopped using remote worship. **Conclusions**: Adults who initiated and continued using remote worship during the COVID-19 pandemic had poorer perceived social support outcomes relative to adults who never used or stopped using remote services. Despite continued engagement with their religious communities, adults participating in worship remotely may have had residual personal, emotional, and instrumental social support needs that remote worship did not mitigate.

## 1. Introduction

The infectious coronavirus disease 2019 (COVID-19) first emerged in December 2019 and has since become a global pandemic, resulting in initial shutdown orders, mask mandates, and ongoing social distancing requirements unlike anything experienced in decades. The impact of COVID-19 has affected every sector of life, including political, economic, and social [1]. In March 2020, when schools and businesses across the U.S. were mandated through local orders to cease all in-person gatherings, many houses of worship voluntarily cancelled in-person meetings, directly impacting the 36% of U.S. adults that attend religious services at least once a week [2]. Consequently, these initial shutdown orders, whether imposed statewide or by religious institutions, drastically disrupted the occurrence of in-person religious worship services. As the pandemic progressed, and even when the shutdown orders were lifted, many religious institutions continued to cancel in-person services, raising the question of how such changes would impact the well-being of congregants. Religious faith is often demonstrated by and fostered through attendance at religious services and is associated with emotional, social, and spiritual health and well-being [3].

Well-supported by extant literature, social support from family, friends, and others in the social network, including religious communities, is critical to overall health and well-being [4]. This support is associated with improvements in perceptions of physical health [5,6] and overall quality of life [7,8]. Social support is also a protective buffer during challenging personal or national crises. For those who consider themselves to be associated with religion, social support can be derived from religious networks that come from that association [9]. Furthermore, those who attend worship services regularly report feeling cared for and valued, largely due to their connection to the worship community [10], and regular attendance is associated with life satisfaction [11]. Social support derived from religious networks also appears to be protective against depressive symptoms and psychological distress [12]. Other research suggests that the perceived support may be less about tangible support received from religious institutions (i.e., instrumental social support) and more linked to the perception of available support and its positive impact on emotional well-being [13]. Research also suggests that individuals’ overall quality of life is positively impacted by social support and the sense of community formed through participation in religious congregations [7,8].

While some past research has validated unidimensional measures of social support, suggesting that social support is a unitary concept (e.g., Oslo Social Support Scale [14]), other researchers have proposed multidimensional measures that reflect specific sources [15] or types of social support, including instrumental, appraisal, emotional, and informational social support [16,17,18]. However, some researchers, including Shakespeare-Finch and Obst (2011) posit that the most important types of social support are instrumental support (i.e., providing tangible, material, or practical support, including provision of childcare, transportation, meals, or financial assistance) and emotional support (i.e., expressing empathy, love, and care) [19]. Indeed, studies have found that both instrumental and emotional forms of social support are associated with increased engagement in healthy behaviors [20] and better physical and mental health [21,22,23].

Given the meaning and impact of attending religious services, it is no surprise that religious institutions almost immediately addressed the suspension of in-person worship services brought about because of stay-at-home orders in many states due to COVID-19 by providing alternative modalities for worship. These alternative modalities included a wide array of virtual services including the use of Facebook, YouTube, and Zoom livestreams [24,25,26,27]. Additionally, religious leaders continued to provide a virtual space for communal gatherings and celebrations of important religious events [28,29,30]. Consequently, there has been a sharp increase of participation in online services across all religious groups, both synchronous and asynchronous [31,32].

Virtual religious services certainly are not new; however, the breadth and scale of virtual religious services in response to COVID-19 has been unprecedented and has continued to expand amidst the ongoing pandemic [33]. Therefore, the many millions living in the U.S. who typically attended religious worship services in-person had to make behavioral adaptations by participating in alternative services to meet their needs for worship. Understanding the impact of the loss of in-person gathering on perceived social support has implications for how religious services are offered during times of crisis [34]. However, the relationship between changes to the use of in-person religious services versus other worship modalities that occurred during the COVID-19 pandemic on the perceived social support of U.S. adults affiliated with a religious group is currently unknown. To address this gap in the literature, the aim of this study was to understand whether changes in the use of remote worship were related to changes in the perceptions of social support among religious adults before and during COVID-19.

## 2. Materials and Methods

### 2.1. Research Design and Participants

This study was a self-reported cross-sectional online survey administered from 15 May to 6 July 2020 using Qualtrics, a web-based research survey tool [35]. A non-probability sample of 501 was recruited from Qualtrics sources such as website intercept recruitment, member referrals, targeted email lists, gaming sites, customer loyalty web portals, permission-based networks, and social media. Participants were recruited from three cities (New York City, NY; Miami, FL; and San Francisco, CA) across the U.S., which were selected due to their diverse populations and high COVID-19 infection rates. Eligible participants were 18 years or older, reported attending religious services at least once a month, identified as either Christian, Jewish, or Muslim, and reported being offered an alternate/remote form of worship. This resulted in an analytical sample of *n* = 461 participants. Institutional Review Board approval was received from Virginia Commonwealth University (HM20019222) and all participants provided informed consent before participating in the study.

### 2.2. Measures

The survey instrument consisted of 49 items. Items in this analysis included:

*Sociodemographics.* These variables included: religious affiliation (Christian, Muslim, and Jewish), age (18–29, 30–49, 50–64, and 65 or older), educational attainment (high school or less, some college, and college or more), gender (male, female, transgender), race/ethnicity (Non-Hispanic white, Black or African American, another race or multiple races, and Hispanic or Latinx), annual household income (<$40,000, $40,000–<$800,000, and $80,000 or more), metropolitan area of residence (Miami, New York City, and San Francisco), and self-reported health status (very poor, poor, neither poor nor good, good, and very good).

*Use of Alternate Worship Services.* Participation in alternative worship services (before and during COVID-19) was based on the question: “Have you participated in any alternative worship services (online, drive-thru, social media, recordings, zoom, etc.)?” Pre-COVID-19 response options were “yes” and “no”. During COVID-19 response options included “yes”, “sometimes”, and “no”.

Changes to the use of alternate worship were trichotomized. Those who continued using remote worship services included individuals with pre-COVID-19 use of remote worship and continued use during the pandemic, including responses of “sometimes” or “yes”. Individuals who started using remote worship services were those with no prior use of remote worship but who began using services during the pandemic, including responses of “sometimes” or “yes”. The last category included people who never used remote worship before or during COVID-19 and those who had previously used remote service before COVID-19, but who never used them during the pandemic.

*Role of Worship Community.* The role of the religious worship community was assessed with three questions: (1) “Do you usually turn to your house of worship’s community for support of your sense of well-being?”; (2) “Do you rely on your house of worship’s community to help you better understand social issues?”; (3) “Do you rely on your house of worship’s community for social support?” Response options were yes, sometimes, and no. An additional question assessed changes due to social distancing: “Has the sense of community between your house of worship’s community members been negatively affected by social distancing policies?” Responses were collected as yes or no.

*Social Support.* Perceived social support was assessed using a slightly modified version of the three-item Oslo Social Support Scale (OSSS-3 [14]), a reliable and validated measure for examining social support. Changes from pre-pandemic to during pandemic levels of social support were assessed by examining changes in individual social support items’ scores and changes in categorical summary scores. While validity studies suggest the use of all three items to measure a unidimensional construct of social support [14], other studies challenge this, and have examined the three items as separate scales [16]. Preliminary evidence also suggests that instrumental and emotional support were differentially related to individuals’ psychological well-being and affect during the COVID-19 pandemic [36]. Moreover, researchers have only recently begun using the OSSS-3 [14] in virtual social settings [37,38]. Thus, changes to single items are examined as well as changes to the overall summary scores.

*Change Scores for Individual Social Support Items.* Two items assessed quantity of social support for personal problems, a form of emotional social support, before vs. during the COVID-19 pandemic: (1) “Before the stay at home orders/recommendations, how many people were so close to you that you could count on them if you had great personal problems?” and (2) “During the stay at home orders/recommendations, how many people were so close to you that you could count on them if you had great personal problems?” Response options included none, 1–2, 3–5, and >5.

Two items assessed the amount of interest and concern felt from other people, which was conceptualized as another indicator of emotional social support, from before versus during the COVID-19 pandemic: (1) “Before the stay at home orders/recommendations, how much interest and concern did people show in what you do?” and (2) “After the stay at home orders/recommendations, how much interest and concern did people show in what you do?” Response options included none, little, uncertain, some, and a lot.

Change to instrumental social support was assessed by asking participants two items about their perceived ease of getting necessary practical help from neighbors before vs. during the COVID-19 pandemic: (1) “Before the stay at home orders/recommendations, how easy was it to get practical help from neighbors if you should need it?” and (2) “After the stay at home orders/recommendations, how easy was it to get practical help from neighbors if you should need it?” Response options included very difficult, difficult, possible, easy, and very easy.

Change scores for each type of social support were calculated by subtracting participants’ responses during COVID from their pre-COVID responses. This resulted in a direction and strength of change that ranged from negative to positive integers.

*Change Scores for Social Support Categorical Summary Score.* Using the scoring system developed and validated by Kocalevent and colleagues (2018), a three-category social support summary score was calculated at each time point (before vs. during pandemic) [14]. Summed values of the items at each time point ranged from three to fourteen, where low scores represented worse social support and higher scores represented greater social support. The thresholds for interpreting scores were as follows: poor social support (3–8), moderate social support (9–11), and strong social support (12–14). Change scores were calculated and indicated the direction and strength of change between categorical summary score from level of social support before the pandemic to level of social support during the pandemic (e.g., poor to strong = +2; poor to moderate = +1; poor to poor = 0). In tables, changes to social support categorical summary scores are labeled as “Change to OSSS-3 summary score”.

### 2.3. Data Analysis

Bivariate analyses of sociodemographic characteristics by changes in individuals’ utilization of remote worship services before and during the COVID-19 pandemic were examined using Chi-squared (χ^2^) tests of independence. The distributions of categorical sociodemographic characteristics and social support items and summary change scores are presented as frequencies and percentages in Table 1 and Table 2, respectively.

Unadjusted multinomial logistic regression models examined the odds of change (increase or decrease; no change was used as the reference category) to three types of social support and overall social support during the COVID-19 pandemic. The independent variable was changes in individuals’ utilization of remote worship services from before to during the COVID-19 pandemic. Results of the multinomial logistic regression are presented as odds ratios and 95% confidence intervals by social support type (i.e., Model 1, Model 2, and Model 3). All analyses were conducted in SPSS Version 28.0.

## 3. Results

Four hundred sixty-one (*n* = 461) individuals completed the online survey and were eligible. The majority (89%) indicated that their religious leader had suspended in-person services. Bivariate analysis of sociodemographic characteristics and changes in individuals’ utilization of remote worship services are presented in Table 1. There were significant differences in faith type, age category, gender, geographic region, household annual income, and health status by change in remote worship service use. In addition, the full distribution of social support item-level and categorical summary score change scores from before to during the COVID-19 pandemic are presented in Table 2.

In unadjusted multinomial regression models, individuals who started using and continued using remote worship services experienced poorer social support than those who never used remote services. Individuals who began using remote worship services during COVID were 62% less likely to report having more people close enough to them that they could count on for significant personal problems (OR: 0.38; 95% CI: 0.19, 0.79) than to report no change in the quantity of people close enough to count on compared to people who never used remote services. In addition, people who began to use remote worship services during COVID were also significantly more likely (OR: 1.77; 95% CI: 1.04, 3.02) to report less perceived ease in getting practical help if needed from their neighbors during COVID than those who never used remote services (see Table 3).

Like those who began to use remote services during the pandemic, individuals who had previously used and continued using remote worship services also reported poorer social support outcomes than those who did not use remote services. Compared to those who never used remote worship services before or during COVID, those who previously used and continued using remote worship services were significantly more likely (OR: 2.23; 95% CI: 1.17, 4.25) to report less perceived ease in getting practical help if needed from their neighbors during COVID than to report no change in ease. Continuing to use remote services was also associated with greater odds (OR: 2.62; 95% CI: 1.24, 5.51) of reporting a decrease of the perceived amount of interest and concern felt from other people during COVID, compared to those who never used remote services (see Table 3).

While item-level social support indicators reflected changes in types of social support based on changes to remote worship services, there were no significant differences in categorical social support summary scores between timepoints. Results of unadjusted multinomial regression models are presented in greater depth in Table 3.

## 4. Discussion

This study assessed how attending alternative religious services related to changes in perceived social support during the early onset of the COVID-19 pandemic. Findings suggest that participating in these services for those who had been regularly attending religious services before the pandemic did not impact perceived social support positively, and in fact, these respondents reported poorer levels of social support compared to those who did not attend the virtual services. The social distancing and initial shut-down orders effectively prevented members of different religious faiths from engaging in traditional, in-person forms of religious practices from which they, to varying degrees, derive spiritual satisfaction, support, a sense of community and/or a sense of well-being [39]. Our study showed that there was indeed an effect on the perceived types of social support for those participating in modified religious services. This behavioral adaptation, from in-person to virtual engagements, including teleworking and virtual services, became the dominant paradigm in the early shutdown phase of the COVID-19 pandemic [40], which coincided with this study period. Large gatherings and social in-person contact very quickly had to be replaced with adaptive behavior relying on virtual modalities. Across the world, religious leaders almost immediately responded to the shutdown orders by offering virtual worship and support services [41,42]. As such, most of this sample switched to virtual services, a modality that prior to COVID, only one-fifth had practiced. Regardless of religious faith, age, educational attainment, gender, race/ethnicity, income, metropolitan area, or health status, every single group increased their adoption of virtual worship service participation.

There are various health-related protective effects associated with being part of a religious community [43,44], and the connection between health benefits of religious involvement and social support is well established [45,46]. In this study, the majority reported that they rely on their religious community for social support and a sense of well-being. More importantly, the majority indicated that they rely on their religious house of worship as a source for a better understanding of social issues. The COVID-19 pandemic has undoubtedly impacted perceived social supports for everyone, including those who attend worship services. The finding that adults who initiated or continued using remote worship during the pandemic reported decreased social support is perhaps not surprising, given that this shift to remote worshiping required a different method of engagement during what was an especially challenging time. This finding is in line with research on the use of social networking sites, indicating that while online networking might enhance informational social support, it may not enhance tangible or instrumental social support [47].

It is also important to note that individuals’ experiences and responses to engaging in online worship and remote religious practice may vary. These alternative forms of worship are still interpersonal exchanges of information whereby two or more individuals engage and appraise their interaction. However, this study did not find a significant relationship between initiating or continuing to use virtual and remote forms of worship with perceived social support. It may be that the lack of the offline, physical networking components that can be so important when participating in activities, such as prayer groups or bible study, may have been lost when shifting to online services, especially at the beginning of the pandemic. There is evidence of this in other work looking at online communities. Differences in online and offline social capital, defined as the resources and benefits available to someone through their interpersonal connections, has been shown to also be associated with perceived social support. However, online social capital is only enhanced if there is offline bonding capital as well, making perceived social support an important pathway [48]. As such, it may be that those who suddenly lost this offline social capital and bonding to others interpersonally impacted their perceived social support more than someone who simply chose not to switch to online services at all. It could also be that COVID-19 caused so many disruptions to life that the cumulative effect may have stunted the effect of virtual social support options, because people in general were not feeling supported during the early days of the pandemic.

Arguably, the COVID-19 pandemic has been a critical public health issue primarily due to its impact, intensity, and unknown duration, and as such, has very direct implications for social and community connectedness and the members’ greater sense of well-being. Within a public health context, religious institutions have demonstrated their critical role in the dissemination and demonstration of social distancing orders and guidelines. Similarly, religious leaders provide guidance on various social and health issues [39,49,50]. There are various public health implications to consider, specifically related to the impact of religious worship services on the sense of community, well-being, and perceived social support for those who choose to attend services on a regular basis [51]. Religious communities are focused on the well-being of their congregants, which can be leveraged to have an even more intentional role in providing varied forms of social support to its members. Furthermore, public health efforts need to include religious leaders and their support staff as key stakeholders more effectively when designing and implementing public health efforts [52,53]. While the pandemic has affected all facets of daily life, the complete impact of which is largely unknown, it is imperative to establish multi-faceted partnerships with religious leaders [54,55] and to leverage the church community as a vital source for addressing congregants’ social support needs, thereby strengthening their role in support of broader public health efforts to emphasize health and wellness [56].

*Study limitations.* Various study limitations need to be noted. First, this was a cross-sectional study using self-rated measures of changes to worship format and social support and was restricted to those of three religious faiths. Therefore, these results may not be generalizable to individuals who are from other religions, do not attend religious services, or consider themselves spiritual and/or practice other forms of worship, including meditation. Furthermore, no data were collected on the level of religiosity prior to the pandemic and remote worship. These data might have provided insights on anticipated usual levels of social support, and other types of social support, such as appraisal support and companionship [57], were not measured in the present study. Second, there are inherent biases in a Qualtrics sample, as individuals with easy access to web-based surveys may have had greater familiarity with technology, and therefore, different pandemic experiences than the general population. For example, most of the sample had college degrees, identified as male, and self-reported their health status as good to very good, which may not be representative of the larger US population. Third, the study data provide a limited yet important snapshot in time, from mid-May to early July 2020. While these were the early months of the pandemic, these data still provide important insights about behavioral adaptation, given that several months later, most in-person religious worship services were still limited.

Another notable limitation is that the three items from the social support scale used (OSSS-3 [14]) have not been validated for interpretation on a single-item basis. In other words, understanding the true association of alternate modalities and social support types may be better understood through using validated, multidimensional measures of social support that use multiple indicators for each form of social support. Another potential limitation is that social support outcomes might have also been related to unmeasured factors such as economic strains due to the lack of childcare, strains on personal relationships, unemployment, mental health, etc. Due to a limited sample size, adjusted multinomial logistic regression models could not be created to help account for some of these factors. Finally, all data were self-reported and based on a moment in time during an evolving pandemic. However, this study has several strengths, including a diverse religious sample from three different states, and data were collected during a very critical time of the pandemic when shutdown orders were in place. This study allows us to provide insights regarding the impact of behavioral adaptations of those who report attending worship services at least once a month on changes to types of social support. Study findings might also suggest that, despite the benefits of remote worship, there may be types of social support that are not supported by virtual settings and are more comprehensively addressed with in-person worship. Future research needs to include a more diverse sample of individuals less often represented in online survey research, including older adults and individuals from racial and ethnic minority groups, and expand to include other faith traditions. Furthermore, the widespread expansion of remote worship during the pandemic may provide further opportunities to examine the impact of pastoral remote support on self-reported social support outcomes, similar to current work on the effects of telemedicine on the patient–provider relationship.

## 5. Conclusions

This pandemic has presented a public health crisis with enormous implications, and has highlighted various issues, including the impact on social support for those who typically attend religious services. As such, public health practitioners need to cultivate and understand the importance of partnerships with religious leaders and of supporting communities where they gather, live, and worship. Finally, while the full effects of the still-evolving COVID-19 pandemic are still unknown, this study provides empirical evidence that social support is multi-faceted, and despite the use and benefits of remote worship services, there are likely various personal, emotional, and instrumental social supports that will require a community-wide collaborative and ongoing response.

## Figures and Tables

**Table 1:** Characteristics of study participants by utilization of remote worship services before and during COVID-19 pandemic among religious adults who were offered remote worship services (*n* = 461).

	Continued Using Remote Worship Services		Started Using Remote Worship Services		Never Used or Stopped Using Remote Worship Services			
	*(n* = 98)		(*n* = 262)		(*n* = 101)			
	*n*	[%]	*n*	[%]	*n*	[%]	χ^2^ (df)	*p*-Value
Faith								
Christian	58	[59.2]	149	[56.9]	57	[56.4]	10.508 (4)	0.033
Jewish	8	[8.2]	56	[21.4]	18	[17.8]		
Muslim	32	[32.7]	57	[21.8]	26	[25.7]		
Age								
18–29 years	14	[14.3]	52	[19.8]	41	[40.6]	80.079 (6)	<0.001
30–49 years	73	[74.5]	88	[33.6]	27	[26.7]		
50–64 years	8	[8.2]	57	[21.8]	18	[17.8]		
65+ years	3	[3.1]	65	[24.8]	15	[14.9]		
Educational Attainment								
High school or less	5	[5.1]	13	[5.1]	9	[9.3]	5.620 (4)	0.229
Associate degree or some college	17	[17.3]	62	[24.1]	26	[26.8]		
Bachelor’s degree or more	76	[77.6]	182	[70.8]	62	[63.9]		
Gender ^a^								
Male	76	[77.6]	162	[61.8]	57	[56.4]	10.852 (2)	0.004
Female	22	[22.4]	100	[38.2]	44	[43.6]		
Race/Ethnicity ^b^								
White, non-Hispanic	76	[77.6]	165	[63.0]	51	[50.5]	17.193 (6)	0.009
Black or AA, non-Hispanic	5	[5.1]	29	[11.2]	15	[14.9]		
Another or multiple races, non-Hispanic	6	[6.1]	19	[7.2]	13	[12.9]		
Hispanic or Latinx	11	[11.2]	49	[18.7]	22	[21.8]		
Geographic Region								
Miami	27	[27.6]	93	[35.3]	44	[43.6]	11.934 (4)	0.018
New York City	56	[57.1]	107	[40.8]	41	[40.6]		
San Francisco	15	[15.3]	62	[23.7]	16	[15.8]		
Household Annual Income								
Less than $40,000	8	[8.2]	49	[18.7]	22	[21.8]	16.196 (4)	0.003
$40,000 to $79,999	16	[16.3]	66	[25.2]	29	[28.7]		
$80,000 or more	74	[75.5]	147	[56.1]	50	[49.5]		
Health Status								
Very good	46	[46.9]	77	[29.4]	33	[32.7]	10.145 (4)	0.038
Good	43	[43.9]	147	[56.1]	55	[54.5]		
Neither, poor, or very poor	9	[9.2]	38	[14.5]	13	[12.9]		
Role of House of Worship								
Sense of Well-being								
Yes	72	[73.5]	121	[46.2]	47	[46.5]	26.049 (4)	<0.001
Sometimes	17	[17.3]	94	[35.9]	29	[28.7]		
No	9	[9.2]	47	[17.9]	25	[24.8]		
Social Issues								
Yes	63	[64.3]	105	[40.1]	41	[40.6]	21.361 (4)	<0.001
Sometimes	18	[18.4]	74	[28.2]	21	[20.8]		
No	17	[17.3]	83	[31.7]	39	[38.6]		
Social Support								
Yes	63	[64.3]	112	[42.7]	42	[41.6]	23.651 (4)	<0.001
Sometimes	14	[14.3}	80	[30.5]	19	[18.8]		
No	21	[21.4]	70	[26.7]	40	[39.6]		
Affected Sense of Community								
Yes	93	[94.9]	217	[82.8]	65	[64.4]	31.449 (2)	<0.001
No	5	[5.1]	45	[17.2]	36	[35.6]		

Notes. AA = African American. df = degrees of freedom. Sense of well-being = usually turn to house of worship’s community for support of sense of well-being. Social Issues = relies on house of worship to help them understand social issues. Social Support = relies on house of worship’s community for social support. Affected Sense of Community = sense of community between house of worship’s community members has been negatively affected by policies of social distancing. ^a^ Gender was dichotomized due to small cell sizes, and one transgender male participant was included in the male gender group and one transgender female participant was included in the female gender group. ^b^ Race/ethnicity was collapsed into four categories due to small cell sizes.

**Table 2:** Distribution of change scores for social support from before to during the COVID-19 pandemic by social support (*n* = 461).

	Change to Quantity of Social Support for Personal Problems, before vs. during COVID-19	Change to Amount of Interest and Concern Felt from other People, before vs. during COVID-19	Change to Perceived Ease of Getting Necessary Practical Help, before vs. during COVID-19	Change to OSSS-3 Summary Score, before vs. during COVID-19
	Possible range: −3 to +3	Possible range: −3 to +3	Possible range: −4 to +4	Possible range: −2 to +2
	% (*n*)	% (*n*)	% (*n*)	% (*n*)
Decrease −4	--	--	2.2% (10)	--
Decrease −3	1.1% (5)	0.7% (3)	2.4% (11)	--
Decrease −2	5.0% (23)	4.6% (21)	10.4% (48)	1.5% (7)
Decrease −1	16.5% (76)	15.2% (70)	20.9% (96)	14.2% (65)
No change +/− 0	67.4% (310)	59.4% (274)	48.5% (223)	57.7% (265)
Increase +1	9.3% (43)	19.1% (88)	12.8% (59)	24.8% (114)
Increase +2	0.7% (3)	0.9% (4)	1.3% (6)	1.7% (8)
Increase +3	0.0% (0)	0.2% (1)	0.9% (4)	--
Increase +4	--	--	0.7% (3)	--

Notes. *n* = 460 for change to social support for personal problems and change to ease of getting necessary practical help because a response was missing for one or both time points. *n* = 459 for change to summary social support score because two responses were missing for one or both time points.

**Table 3:** Unadjusted multinomial logistic regression models for odds of increase or decrease for three types of social support and categorical summary social support score during the COVID-19 pandemic (*n* = 461).

Model 1. ODDS of Increased or Decreased Social Support for Personal Problems								
	More People Close Enough to Count on for Personal Problems				Fewer People Close Enough to Count on for Personal Problems			
	OR	(95% CI)	Wald(df)	*p*-value	OR	(95% CI)	Wald(df)	*p*-Value
Change to remote worship use								
Continued using remote	0.71	(0.31, 1.66)	0.624 (1)	0.711	1.21	(0.63, 2.32)	0.317 (1)	0.573
Started using remote	0.38	(0.19, 0.79)	6.798 (1)	0.009	0.70	(0.40, 1.22)	1.596 (1)	0.206
Never used or stopped using remote	1.00	(ref.)	--	--	1.00	(ref.)	--	--
Model 2. Odds of increased or decreased level of interest and concern from other people								
	Feeling greater interest and concern from other people				Feeling lesser levels of interest and concern from other people			
	OR	(95% CI)	Wald(df)	*p*-value	OR	(95% CI)	Wald(df)	*p*-value
Change to remote worship use								
Continued using remote	1.80	(0.88, 3.69)	2.576 (1)	0.108	2.62	(1.24, 5.51)	6.383 (1)	0.012
Started using remote	1.28	(0.70, 2.34)	0.628 (1)	0.428	1.71	(0.89, 3.28)	2.574 (1)	0.109
Never used or stopped using remote	1.00	(ref.)	--	--	1.00	(ref.)	--	--
Model 3. Odds of increased or decreased perceived ease of getting necessary practical help from neighbors								
	Easier to get necessary practical help from neighbors				Harder to get necessary practical help from neighbors			
	OR	(95% CI)	Wald(df)	*p*-value	OR	(95% CI)	Wald(df)	*p*-value
Change to remote worship use								
Continued using remote	1.98	(0.93, 4.20)	3.134 (1)	0.077	2.23	(1.17, 4.25)	5.942 (1)	0.015
Started using remote	0.89	(0.46, 1.72)	0.127 (1)	0.722	1.77	(1.04, 3.02)	4.473 (1)	0.034
Never used or stopped using remote	1.00	(ref.)	--	--	1.00	(ref.)	--	--
Model 4. Odds of increased or decreased categorical summary social support score								
	Lower social support					Higher social support		
	OR	(95% CI)	Wald (df)	*p*-value	OR	(95% CI)	Wald (df)	*p*-value
Change to remote worship use								
Continued using remote	1.73	(0.74, 4.05)	1.593 (1)	0.207	1.28	(0.67, 2.42)	0.562 (1)	0.454
Started using remote	1.73	(0.84, 3.56)	2.212 (1)	0.137	1.03	(0.61, 1.78)	0.015 (1)	0.904
Never used or stopped using remote	1.00	(ref.)	--	--	1.00	(ref.)	--	--

Notes. OR = odds ratio; df = degrees of freedom; CI = confidence interval. Reference group for odds of increased or decreased social support is no change to that type of social support relative to before the COVID-19 pandemic. Changes to categorical summary social support score thresholds were determined using cut-points determined by Kocalevent et al. (2018) [14].

## Data Availability

The data that support the findings of this study are available from the corresponding author, M.M., upon reasonable request.
